# Nanozymes: Versatile Platforms for Cancer Diagnosis and Therapy

**DOI:** 10.1007/s40820-022-00828-2

**Published:** 2022-04-06

**Authors:** Xiaodong Zhang, Xiaokai Chen, Yanli Zhao

**Affiliations:** Division of Chemistry and Biological Chemistry, School of Physical and Mathematical Sciences, Nanyang Technological University, 21 Nanyang Link, Singapore, 637371 Singapore

**Keywords:** Cancer theranostics, Catalytic therapy, Enzyme mimics, Nanozymes, Smart nanomedicine

## Abstract

Natural enzymes usually suffer from high production cost, ease of denaturation and inactivation, and low yield, making them difficult to be broadly applicable. As an emerging type of artificial enzyme, nanozymes that combine the characteristics of nanomaterials and enzymes are promising alternatives. On the one hand, nanozymes have high enzyme-like catalytic activities to regulate biochemical reactions. On the other hand, nanozymes also inherit the properties of nanomaterials, which can ameliorate the shortcomings of natural enzymes and serve as versatile platforms for diverse applications. In this review, various nanozymes that mimic the catalytic activity of different enzymes are introduced. The achievements of nanozymes in different cancer diagnosis and treatment technologies are summarized by highlighting the advantages of nanozymes in these applications. Finally, future research directions in this rapidly developing field are outlooked.

## Highlights

- This review introduces nanozymes that exhibit different enzymatic activities and emphasizes the advantages of nanozymes over natural enzymes.
- The roles of nanozymes in different cancer diagnostic and therapeutic technologies are summarized, explained by representative examples.
- The potential challenges of nanozyme-based cancer theranostics are outlined, and future research directions are outlooked.

## Introduction

Cancer is a notorious disease worldwide with nearly 20 million new cases and 10 million deaths in 2020 [1]. Due to the rapid increase in cancer incidence and mortality, novel remedies and therapeutic agents are in urgent need. Additionally, early diagnosis of cancer with high sensitivity and accuracy is of great importance. As a frontier science, nanotechnology has become a cutting-edge tool for solving a variety of world-class scientific issues including cancer theranostics. Compared to conventional medicine, nanoparticle formulations usually have improved biodistribution and enhanced accumulations in tumors, thus exhibiting elevated therapeutic efficiencies with reduced side effects [2–5]. Moreover, taking advantage of their unique properties, nanoparticles can function as imaging agents for tumor tracking, remodel the tumor microenvironment, and even serve as drug-free therapeutics, providing versatile platforms for cancer diagnosis and therapy [6–11].

On the other hand, enzymes are biocatalysts that mediate a wide variety of reactions during biological processes such as signal transduction, metabolism, and digestion. In particular, specific enzymes are closely related to diseases. For instance, proteolytic enzymes (e.g., matrix metalloproteinases, serine proteases, and cysteine cathepsins) have been demonstrated to facilitate tumor progression [12]. In addition to vital roles in organisms, enzymes are broadly applicable for commercial purposes due to their high specificity and efficiency [13]. As an example, protease, amylase, lipase, and mannanase are auxiliary components of detergents for decontamination of corresponding substrates [14]. Nevertheless, the widespread application of enzymes is severely hindered by several limitations. First, enzymes are proteins or RNA that can be easily degraded by protease and ribonuclease respectively, making them difficult to store and transport. Second, the catalysis specificity of enzymes relies on their unique three-dimensional structures that are sensitive to the environment. As a result, when exposed to extreme conditions such as strong acid/alkali and high temperature, enzymes are easily denatured and inactivated. Third, enzymes are usually extracted from living cells, leading to costly and cumbersome separation and purification processes.

**Fig. 1:**
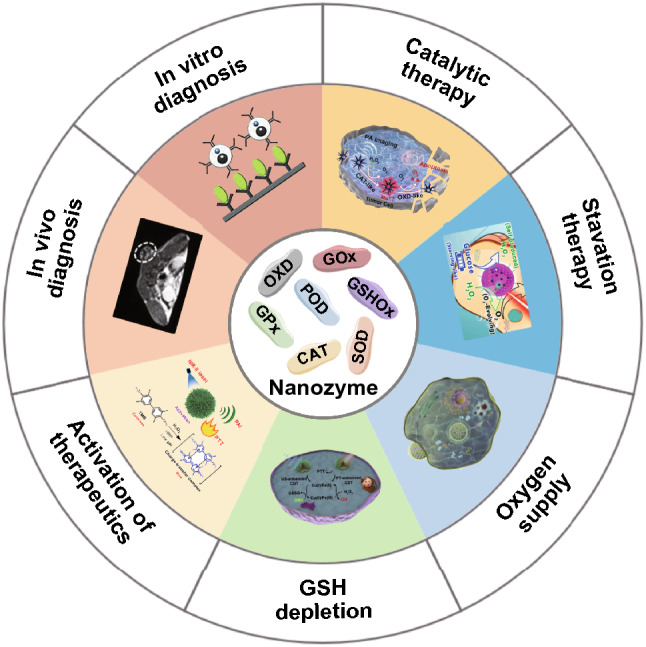
Schematic illustration indicating the applications of nanozymes in cancer diagnosis in vitro (reproduced with permission from Ref. [33]. Copyright 2013, WILEY–VCH GmbH) and in vivo (reproduced with permission from Ref. [34]. Copyright 2020, WILEY–VCH GmbH), and cancer therapy including catalytic therapy (reproduced with permission from Ref. [35]. Copyright 2019, American Chemical Society), starvation therapy (reproduced with permission from Ref. [36]. Copyright 2015, American Chemical Society), GSH depletion (reproduced with permission from Ref. [37]. Copyright 2021, American Chemical Society), oxygen supply (reproduced with permission from [38]. Copyright 2020, Nature Publishing Group), and activation of therapeutics (reproduced with permission from Ref. [39]. Copyright 2019, American Chemical Society)

To solve these problems, the development of artificial enzymes is considered a potential method. Particularly, nanozymes with both enzyme-mimicking capability and nanometer size have emerged as a rising star in the field of artificial enzymes [15–17]. Nanozymes are defined as nanomaterials that can catalyze chemical reactions involving enzyme substrates under physiological conditions by following enzyme kinetics [18]. Similar to natural enzymes, nanozymes possess high catalytic activities and can accelerate biochemical reactions. By featuring the nature of nanomaterials, nanozymes usually have good stability, low production cost, and simple preparation/purification procedures [19, 20]. Up to now, a large number of nanomaterials such as metal nanoparticles, metal oxide nanoparticles, carbon-based nanomaterials, and metal–organic frameworks have been proven to be able to behave like natural enzymes [21–24]. Furthermore, these nanozymes show distinct advantages in biomedical applications, especially cancer theranostics [25–32]. In this review, we introduce the role of enzymes in biological systems and summarize which nanomaterials have enzyme-mimicking capability. Then, the recent representative advances of nanozymes [33–39] in different cancer diagnostic and therapeutic methods are highlighted (Fig. 1).

## Classification of Nanozymes

**Fig. 2:**
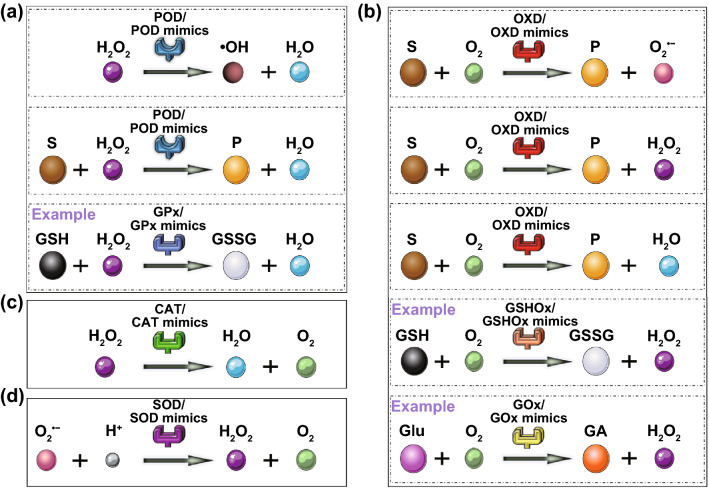
Schematic presentation showing the catalysis reaction mediated by different oxidoreductases and their mimics including **a** POD/POD mimics and a typical kind of POD/POD mimics (glutathione peroxidase, GPx), **b** OXD/OXD mimics and two typical kinds of OXD/OXD mimics (glutathione oxidase, GSHOx; glucose oxidase, GOx), **c** CAT/CAT mimics, and **d** SOD/SOD mimics. The chemical species upon which enzymes act are termed substrates (S), and the molecules obtained from enzyme-catalyzed reactions are termed products (P). GSH, glutathione; GSSG, glutathione disulfide; Glu, glucose; GA, gluconic acid

Generally, according to their different mechanisms, enzymes can be classified into seven types, including oxidoreductases, transferases, hydrolases, lyases, isomerases, ligases, and translocases. Most nanozymes can mimic the activity of oxidoreductases such as peroxidase (POD), oxidase (OXD), catalase (CAT), and superoxide dismutase (SOD), and a small part of them have a similar catalytic ability with hydrolases or others [21]. Since almost all nanozymes reported for cancer theranostics possess oxidoreductase-like activities, we mainly introduce this type of nanozymes in this section (Fig. 2).

### POD-like Nanozymes

PODs typically catalyze the oxidation of the substrates with the consumption of H_2_O_2_ or organic peroxides (Fig. 2a). Most natural PODs are ferric heme proteins that can activate H_2_O_2_ and generate intermediate species with high valence, capable of abstracting electrons from different substrates. As a result, a large number of iron-based nanomaterials have been found to have POD-mimicking ability.

**Fig. 3:**
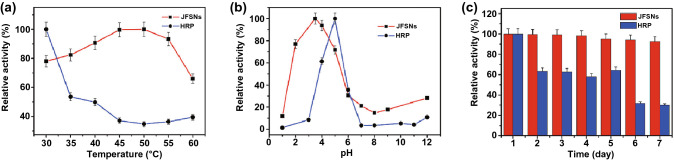
**a**–**c** pH-, temperature-, and time-dependent POD activities of the nanozyme JFSNs and the natural enzyme HRP. Reproduced with permission from Ref. [41]. Copyright 2015, American Chemical Society

For instance, the pioneering work by Gao and coworkers in 2007 indicated that Fe_3_O_4_ nanoparticles possessed an intrinsic catalytic activity toward classical POD substrates including 3,3,5,5-tetramethylbenzidine (TMB), di-azo-aminobenzene (DAB), and o-phenylenediamine (OPD) [40]. Similar to Fe_3_O_4_ nanoparticles, other iron-containing nanomaterials can also serve as POD mimics, in which iron usually exists in the form of Fe_2_O_3_ [41], iron chalcogenides [42], Prussian blue [43], single iron site (i.e., Fe–N–C [44]), hemin [45], and other compounds [27]. As an example, Lu and coworkers prepared Janus *γ*-Fe_2_O_3_/SiO_2_ nanocomposites (JFSNs), whose catalytic activity was higher and more stable in a broad range of pH and temperature than that of the natural enzyme horseradish peroxidase (HRP) (Fig. 3) [41]. JFSNs had a lower Michaelis constant (*K*_m_) value and a higher *V*_max_ value than natural HRP when TMB served as the substrate, indicating that JFSNs possessed a higher affinity and a stronger enzymatic activity (Table 1). Geng et al. developed the biomimetic nanozymes formed by the coassembly of hemin and amphiphilic 9-fluorenylmethyloxycarbonyl (Fmoc)-*L*-histidine (FH) [45]. The size and morphology of the nanozymes could be tuned by adjusting the ratio of the two components, further tailoring their POD-mimicking activities.

**Table 1:** POD-like catalytic activities of natural HRP and JFSNs

Sample	Substrate	*K*_*m*_ (mM)	*V*_max_ (mM min^–1^)
Natural HRP	TMB	5.90	0.11
	H_2_O_2_	0.63	1.35
JFSNs	TMB	3.05	0.25
	H_2_O_2_	965.98	769.65

Furthermore, the nanomaterials that contain other transition metals including V [46, 47], Zn [48], Co [49], Mn [50], Mo [51], W [52], Cu [53], Au [54], Ag [55], Pt [56], Pd [57], Ir [58], Os [59], and Ru [60] can also behave as PODs. André et al. found that V_2_O_5_ nanowires with intrinsic POD-like activity could catalyze the oxidation of the POD substrates including 2,2-azino-bis(3-ethylbenzothiazoline-6-sulfonic acid) (ABTS) and TMB with the help of H_2_O_2_ [46]. Their *K*_m_ values for ABTS oxidation and H_2_O_2_ at pH 4.0 were determined to be 2.9 and 0.4 μM, respectively, which are remarkably lower than those of the natural enzyme HRP and vanadium-dependent haloperoxidase (V-HPO). The above results indicated that V_2_O_5_ nanowires possessed a higher affinity to the POD substrates. In another work, Ghosh and coworkers prepared four kinds of GPx-mimicking V_2_O_5_-based nanomaterials with different morphologies including nanowires, nanosheets, nanoflowers, and nanospheres [61]. They revealed that the GPx-like activities of the nanozymes were not only related to their size and morphology, but also depended on the crystal faces exposed on their surface.

Although some natural enzymes contain metal ions as cofactors, metal elements are not required by most other enzymes to exhibit catalytic activity [62]. In this manner, nonmetallic element-based nanomaterials, especially carbon-based nanomaterials, can also be POD mimics [63–67]. Song and coworkers found that single-walled carbon nanotubes had the intrinsic POD-like property and could catalyze the formation of oxidized TMB to produce a color change. This phenomenon was successfully utilized for detecting disease-associated single-nucleotide polymorphism without further labeling [64]. Similarly, they also observed that graphene oxide (GO) nanosheets could mimic the activity of PODs [65]. Taking advantage of this property, an easy and cheap colorimetric approach for glucose detection with high sensitivity and selectivity was built by GO nanozymes and GOx. As zero-dimensional carbon nanomaterials, carbon/graphene quantum dots can also have a similar activity to PODs. Zhong et al. prepared wood soot-derived carbon quantum dots to catalyze the TMB oxidation with the aid of H_2_O_2_ [66]. Recent work by Wang and coworkers uncovered that the POD-mimicking enzymic activity of specific oxygenated groups enriched graphene quantum dots was much higher than that of classic graphene quantum dots, because the former had more carbonyl and carboxyl groups acting as catalytic sites and binding sites [67].

### OXD-like Nanozymes

OXDs are usually involved in oxidation–reduction reactions at the expense of molecular oxygen (O_2_), which is reduced to H_2_O_2_ or H_2_O (Fig. 2b). In 2004, Comott et al. reported that small-sized gold nanoparticles (AuNPs) could promote the transformation of glucose into gluconate, behaving like GOx [68]. Since then, ultrasmall nanomaterials based on noble metals such as Cu [69], Au [70, 71], Ag [72], Pt [73, 74], and Ir [75] have been proven to have OXD-mimicking ability. For example, Luo et al. designed a self-limiting system using GOx-like AuNPs as both seeds and catalysts [71]. Typically, AuNPs with a size of 13 nm showed a slightly lower affinity but a higher reaction rate for glucose than that of natural GOx (Table 2). Cui et al. synthesized Ir nanoparticles with a small diameter of 2.5 ± 0.5 nm, which could catalyze the oxidation of TMB to form a blue product in the aerobic environment [75]. Taking advantage of their OXD-like property, Ir nanoparticles could be used for detecting dissolved oxygen. In another work, Wang and coworkers prepared GSHOx-like nanozymes based on single-site copper (copper hexacyanoferrate, Cu-HCF), which could catalyze the generation of H_2_O_2_ when consuming glutathione (GSH) [76].

**Table 2:** GOx-like catalytic activities of natural GOx and AuNPs (13 nm)

Sample	Substrate	*K*_*m*_ (mM)	*V*_max_ (μM s^–1^)	*k*_cat_ (s^–1^)
Natural GOx	Glucose	4.87	0.69	9.71
AuNPs (13 nm)	Glucose	6.97	0.63	18.52

**Fig. 4:**
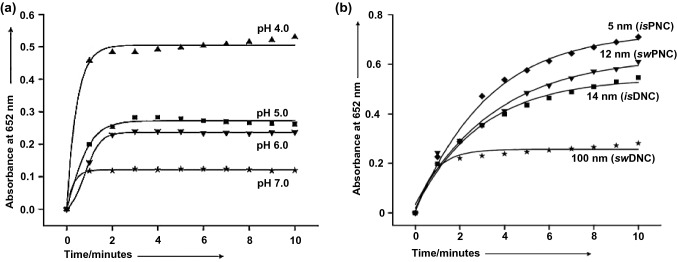
**a–b** pH- and size-dependent OXD-like activities of nanoceria indicated by TMB. Reproduced with permission from Ref. [77]. Copyright 2009, WILEY–VCH GmbH

Cerium oxide nanoparticles (also called nanoceria or ceria nanoparticles) are also widely investigated nanozymes with OXD-like catalytic capability [77–81]. Asati et al. reported that nanoceria promoted the oxidation of different substrates when placed in acidic conditions (Fig. 4a), exhibiting their inherent OXD-like activity [77]. Moreover, it was demonstrated that the activity of nanoceria increased upon decreasing the size (Fig. 4b).

Besides inorganic nanomaterials, organic nanoassemblies can also be OXD mimics. For example, Li and coworkers found that phthalocyanine could be assembled with FH to fabricate photooxidase-like nanovesicles [82]. The self-aggregation of phthalocyanine was reduced after the formation of nanovesicles, thus enhancing its photosensitization activity and photostability. The nanovesicles behaved like OXDs under light irradiation for improved dopamine photooxidation.

### CAT-like Nanozymes

CAT is a typical biological catalyst that exists in almost all living systems and fosters the decomposition of H_2_O_2_ into H_2_O and O_2_ (Fig. 2c). The natural enzyme CAT comprises four iron-containing heme moieties that endow it to have strong interaction with the substrate H_2_O_2_.

**Fig. 5:**
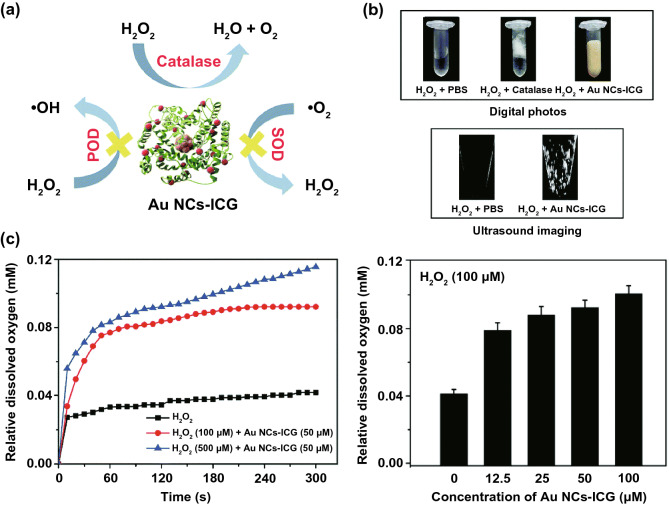
**a** Schematic illustration for Au NCs-ICG with high CAT-like activity. **b** Bright and ultrasound images of H_2_O_2_ after different treatments. **c** O_2_ production ability of Au NCs-ICG in the presence of H_2_O_2_. Reproduced with permission from Ref. [86]. Copyright 2020, The Royal Society of Chemistry

To mimic the enzymatic activity of CAT, many metal-containing and carbon-based nanomaterials have been developed [83–91]. For instance, Dan and coworkers designed indocyanine green-loaded ultrasmall gold nanoclusters (Au NCs-ICG) that had a similar catalytic capability to CAT but not POD or SOD (Fig. 5a, b) [86]. The as-prepared Au NCs-ICG exhibited high substrate affinity (*K*_m_ ≈ 2.02 mM) and superior CAT-like activity (*V*_max_ ≈ 4.55 × 10^−3^ mM s^−1^) (Fig. 5c). Li et al. reported that Fe^3+^ could drive the self-assembly of Fmoc-protected cysteine [87]. After entering the cells, the nanoassemblies would be disassembled, releasing Fe^3+^ for catalyzing the decomposition of H_2_O_2_ into O_2_. Similarly, Fe^3+^ and adenosine monophosphate (AMP) were reported to be able to form coordination nanoparticles (CPs) with strong CAT-mimicking activity [92]. Compared with the natural CAT, the Fe^3+^/AMP CPs had a lower *K*_m_ value and a higher *k*_cat_/*K*_m_ ratio (Table 3), indicating that CPs possessed higher affinity and catalytic efficiency for degrading H_2_O_2_. In another work, gas bubbles were observed when graphene oxide quantum dots were incubated with H_2_O_2_, indicating that the graphene oxide quantum dots had similar functions with CAT to catalytically decompose H_2_O_2_ to generate O_2_ [84]. Furthermore, the authors found that the enzymatic activity of 100 µg L^−1^ graphene oxide quantum dots was comparable to that of 4 U L^−1^ natural enzyme CAT, indicating the high activity of the graphene oxide quantum dots.

**Table 3:** CAT-like catalytic activities of natural CAT and Fe^3+^/AMP CPs

Sample	Substrate	*K*_*m*_ (mM)	*V*_max_ (μM s^–1^)	*k*_cat_/*K*_*m*_ (mL s^–1^ g^–1^)
Natural CAT	H_2_O_2_	249.4 ± 1.2	3.0 ± 0.2	0.12
Fe^3+^/AMP CPs	H_2_O_2_	112.2 ± 0.8	2.4 ± 0.1	0.22

### SOD-like Nanozymes

SOD catalyzes the dismutation of the superoxide radical (O_2_^·−^) into O_2_ and H_2_O_2_ (Fig. 2d), constituting an important antioxidant defense against oxidative stress in the body. Natural SODs usually belong to metalloproteins, and can be divided into three types depending on the protein fold and the metal cofactor, including the Cu/Zn type (which contains Cu and Zn), Fe and Mn types (which contains Fe or Mn), and the Ni type.

Nanomaterials containing these metal elements have been constructed with SOD-like activity [93–100]. For instance, Korschelt et al. synthesized water-dispersible glycine-functionalized Cu(OH)_2_ nanoparticles (Gly-Cu(OH)_2_ NPs) whose O_2_^·−^ decomposition ability was demonstrated using iodonitrotetrazolium chloride as an O_2_^·−^ sensitive indicator [96]. Moreover, Gly-Cu(OH)_2_ NPs had stronger SOD-like activity than the natural enzyme CuZn SOD and bulk Cu(OH)_2_ (Table 4). In another work, Mu et al. utilized hierarchical NiO nanoflowers to scavenge O_2_^·−^ and generate O_2_ with high efficiency, demonstrating good SOD-mimicking enzymatic activity of the NiO nanoflowers [94].

**Table 4:** SOD-like catalytic activities of natural CuZn SOD, Gly-Cu(OH)_2_ NPs, and bulk Cu(OH)_2_

Sample	Substrate	IC_50_ (µM)	IC_50_ per active site (µM)	*k* (M^–1^ s^–1^)	*k* per active site (M^–1^ s^–1^)
Natural CuZn SOD	O_2_^·−^	9.82 × 10^–3^	9.82 × 10^–3^	1.98 × 10^9^	1.98 × 10^9^
Gly-Cu(OH)_2_ NPs	O_2_^·−^	2.25 × 10^–14^	5.02 × 10^–10^	8.72 × 10^14^	3.91 × 10^10^
Bulk Cu(OH)_2_	O_2_^·−^	–	–	–	–

Other nanomaterials also show distinct SOD-mimicking capability. Korsvik and coworkers reported that ceria nanoparticles with a high ratio of Ce^3+^/Ce^4+^ had a strong SOD-mimicking ability, which was because the high Ce^3+^/Ce^4+^ ratio in nanoparticles correlates with higher oxygen and electron vacancy [101]. Samuel et al. found that nanomolar concentrations of hydrophilic carbon clusters were able to rapidly scavenge micromolar to millimolar concentrations of toxic O_2_^·−^, and the major catalytic products were proven as H_2_O_2_ and O_2_, making the nanosystem a suitable biomimetic SOD [102].

## Nanozymes for Cancer Diagnosis

To fight against cancer, early diagnosis is a key to successful treatment. Delayed diagnosis has a greatly negative impact on the survival rate, which is illustrated by the example of patients with renal cancers. The survival rate of patients with early-stage renal cancer is up to 99%, which is much higher than that of patients with second or higher stage renal cancer (16%) [103]. Hence, the main benefit of early diagnosis is the augmentation in the proportion of survived patients with cancers, and developing early diagnosis methods with high sensitivity and selectivity is highly significant. On the other hand, selective tumor imaging with high accuracy is of vital importance for different cancer remedies such as surgery, radiotherapy (RT), phototherapy, and sonodynamic therapy (SDT). Featuring the merits of superior catalytic activity, low cost, high stability, and multifunctionality, nanozymes have been successfully utilized for the detection of cancer-related genes, molecules, and cells, and can serve as probes for accurate imaging due to their intrinsic properties of nanomaterials (Table 5).

**Table 5:** Nanozymes for cancer diagnosis

Nanozyme	Activity	Substrate	Method	Application	References
PSMOF	OXD	TMB	Colorimetry	GSH detection	[105]
Antibody-conjugated Fe_3_O_4_/Pt nanocomposites	POD	TMB	Colorimetric immunoassay	HER2 protein detetion	[33]
FeTIR	POD	TMB	PA	In vivo tumor imaging	[34]
AuVCs	POD	HAuCl_4_ and H_2_O_2_	Lateral flow plasma sensing	GSH detection	[106]
Pt@mSiO_2_	POD	TMB	Colorimetry	BRCA1 gene detection	[107]
5mc-MIO	POD	TMB	Colorimetry and electrochemistry	Detection of global DNA methylation	[108]
PdCu@HRP	POD	TMB	Colorimetry	CEA glycoprotein detection	[109]
GSF@AuNPs	POD	TMB	Colorimetry	Cancer cell detection	[110]
HccFn(Co_3_O_4_)	POD	DAB	Immunohistochemical assay	HCC cell detection	[111]

### In Vitro Cancer Diagnosis

#### GSH Detection

GSH is a ubiquitous tripeptide in which glutamate and glycine are linked by a cysteine residue. It is an important antioxidant and the most abundant low-molecular thiol in living systems, whose concentration in cancer cells is normally higher than that in normal ones [104]. Up to now, a series of GSH detection systems based on nanozymes have been developed, which can realize the distinguishment of cancer cells from normal ones.

**Fig. 6:**
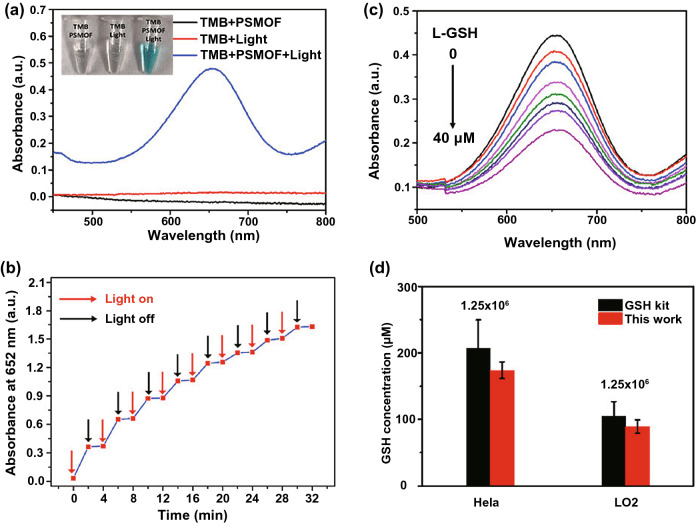
**a** UV−Vis absorption spectra and photo of different samples as indicated. **b** Light-controlled OXD-mimicking ability of PSMOF. **c** UV– Vis absorption spectra of PSMOF-oxidized TMB incubated with different concentrations of GSH. **d** Intracellular GSH concentrations measured by the developed system and a commercial GSH detection kit. Reproduced with permission from Ref. [105]. Copyright 2019, American Chemical Society

For instance, Liu and coworkers designed a photosensitized metal–organic framework (PSMOF) with photo-controlled OXD-mimicking ability [105]. The PSMOF effectively catalyzed the oxidation of TMB under visible light irradiation, obtaining the product (oxidized TMB) with a strong absorption peak at 652 nm (Fig. 6a, b). Interestingly, the absorbance of oxidized TMB at 652 could decrease with the increasing concentration of GSH (Fig. 6c), making the system a colorimetric probe for GSH detection. Moreover, the system was used to analyze the GSH level in the lysates of normal (LO2) and cancer (HeLa) cells. The GSH concentration in cancer cells was determined as about 2-folds higher than that in normal ones, which was in accordance with the results measured by a commercial GSH detection kit (Fig. 6d). In another work, Pang et al. constructed a lateral flow plasmonic biosensor (LFPB) based on gold-viral biomineralized nanoclusters (AuVCs) with POD-like activity [106]. In the presence of HAuCl_4_ and H_2_O_2_, the AuVCs could catalyze the formation of AuNPs, generating colored patterns. Moreover, GSH could inhibit the POD-like activity of the AuVCs and further hinder the formation of AuNPs. Taking advantage of this phenomenon, the LFPB was successfully used for GSH detection. In addition, the authors proved that the intracellular GSH concentration was closely related to the drug resistance of cancer cells, and higher GSH levels were detected in cancer cells with high drug resistance than those with low drug resistance.

#### Detection of Other Biomarkers

Cancer biomarkers are biological molecules produced by the body or tumor in a person with cancer. In addition to GSH, biomarkers can be DNA, RNA, or proteins that are specific to the tumor. With the excellent enzyme-mimicking ability, nanozymes have been used for detecting different biomarkers.

**Fig. 7:**
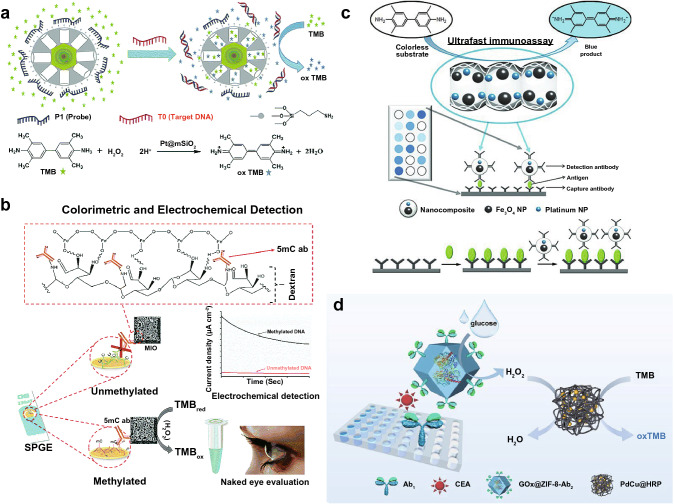
**a** Scheme illustrating the mechanism of POD-like Pt@mSiO_2_ for DNA detection. Reproduced with permission from Ref. [107]. Copyright 2014, The Royal Society of Chemistry. **b** Schematic representation for the detection of global DNA methylation using 5mc-MIO. Reproduced with permission from Ref. [108]. Copyright 2018, The Royal Society of Chemistry. **c** Colorimetric immunoassay for antigen (e. g., HER2) detection based on the antibody-conjugated nanocomposites with POD-like ability. Reproduced with permission from Ref. [33]. Copyright 2013, WILEY–VCH GmbH. **d** Schematic illustration for the catalytic cascade detection of CEA by PdCu@HRP. Reproduced with permission from Ref. [109]. Copyright 2021, American Chemical Society

Wang et al. designed Pt@mesoporous silica (Pt@mSiO_2_) core–shell nanoparticles whose POD-like ability was blocked after probe DNA (P1) adsorption (Fig. 7a) [107]. After incubation with the complementary single-stranded target DNA (T0), the POD-like enzymatic ability of Pt@mSiO_2_ would be recovered, thus catalyzing the color reaction of TMB. Based on this system, a single-base mutation associated with the breast cancer gene BRCA1 was successfully identified. DNA methylation is related to the malignant phenotype of colorectal adenomas. To detect the global methylation with high sensitivity, Bhattacharjee et al. extracted the target DNA and obtained single-stranded DNA (ssDNA) after denaturation followed by direct adsorption onto the surface of a bare screen-printed gold electrode (SPGE) [108]. They also designed the POD mimetic activity of 5-methylcytosine antibody (5mc) functionalized mesoporous iron oxide (5mc-MIO) nanoparticles, in which 5mc was used to recognize the methylcytosine groups in DNA on the SPGE. 5mc-MIO could catalyze the TMB solution in the presence of H_2_O_2_ to give the colorimetric and electrochemical detection of DNA methylation (Fig. 7b). The method could detect as low as 10% difference in the global DNA methylation level in synthetic samples and cell lines with good reproducibility and specificity.

Besides DNA, protein-based cancer biomarkers can be detected using nanozymes. Kim et al. prepared antibody-conjugated nanocomposites with POD-like ability due to the presence of Fe_3_O_4_ and Pt nanoparticles in the nanocomposites [33]. Taking advantage of the enzyme-linked immunosorbent assay (ELISA) technique, they developed a rapid, robust, and convenient antigen detection system based on the nanocomposites (Fig. 7c). Human epidermal growth factor receptor 2 (HER2), a well-known breast cancer marker, could be detected using the colorimetric immunoassay with a limit of detection of 1.5 ng/mL, which was much lower than its clinical cutoff value (15 ng mL^−1^). In another work, POD-like PdCu hydrogel nanozymes with a hierarchically porous structure were employed to load the natural enzyme HRP to obtain PdCu@HRP [109]. In addition to the improved stability and reusability, PdCu@HRP showed higher enzymatic activities than HRP and PdCu hydrogels alone due to the synergistic effect. Combined with the GOx-encapsulated zeolitic imidazolate framework-8 conjugated with antibody (GOx@ZIF-8-Ab_2_), the carcinoembryonic antigen (CEA), a cell surface glycoprotein related to lung, liver, pancreas, breast, cervix, and prostate cancer, was detected using colorimetric biosensing via catalytic cascade reactions (Fig. 7d), whose sensitivity was more than 6 times higher than that of HRP-based ELISA.

#### Cancer Cell Detection

Conjugation with targeting molecules on the surface can endow the nanozymes with tumor-targeting capability. Combined with their excellent enzyme-mimicking properties, nanozyme-based systems can realize selective cancer cell detection.

**Fig. 8:**
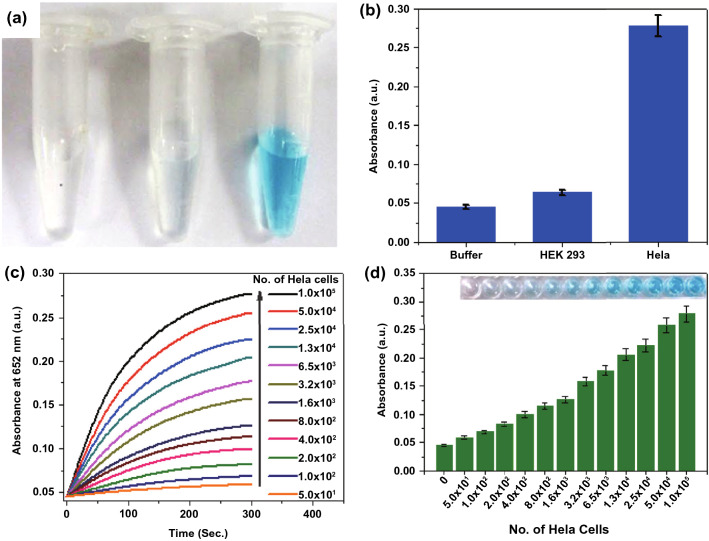
**a** Typical photographs for cell detection using GSF@AuNPs. **b** Selective cancer cell detection using GSF@AuNPs. **c** Time-dependent absorbance at 652 nm when GSF@AuNPs and TMB were incubated with different numbers of HeLa cells. **d** Absorbance at 652 nm when GSF@AuNPs and TMB were incubated with different numbers of HeLa cells for 300 s. Reproduced with permission from Ref. [110]. Copyright 2015, American Chemical Society

For instance, Maji and coworkers prepared the hybrids (GSF@AuNPs) composed of Au nanoparticles, mesoporous silica-coated nanosized reduced graphene oxide, and folic acid for cancer cell targeting [110]. On basis of the unprecedented POD-like activity, the hybrid could serve as a colorimetric nanoprobe for selective, quantitative, and fast detection of cancerous HeLa cells, but not normal HEK 293 cells (Fig. 8). Similarly, ferritin-based cobalt nanozymes (HccFn(Co_3_O_4_)) with intrinsic POD-like activity were modified with the SP94 peptide that can specifically bind to hepatocellular carcinoma (HCC) cells [111]. The nanozymes could catalyze the transformation of the colorless POD substrate diaminobenzidine (DAB) into dark-brown products to visualize HCC tumor tissues with a sensitivity of 63.5% and specificity of 79.1%, which was comparable with that of the clinically used HCC-specific marker.

### In Vivo Cancer Diagnosis

Inheriting the unique physical and chemical properties of nanomaterials, nanozymes have been widely employed for in vivo tumor imaging through different imaging techniques such as fluorescence [112, 113], ultrasound [114], magnetic resonance (MR) [38, 112, 114–116], photoacoustic (PA) [35, 113, 116–120], photothermal (PT) [115–117, 119, 120], and computed tomography (CT) imaging [115, 118, 119]. Moreover, combined with their enzyme-mimicking capability, smart cancer diagnosis systems can be developed based on nanozymes.

**Fig. 9:**
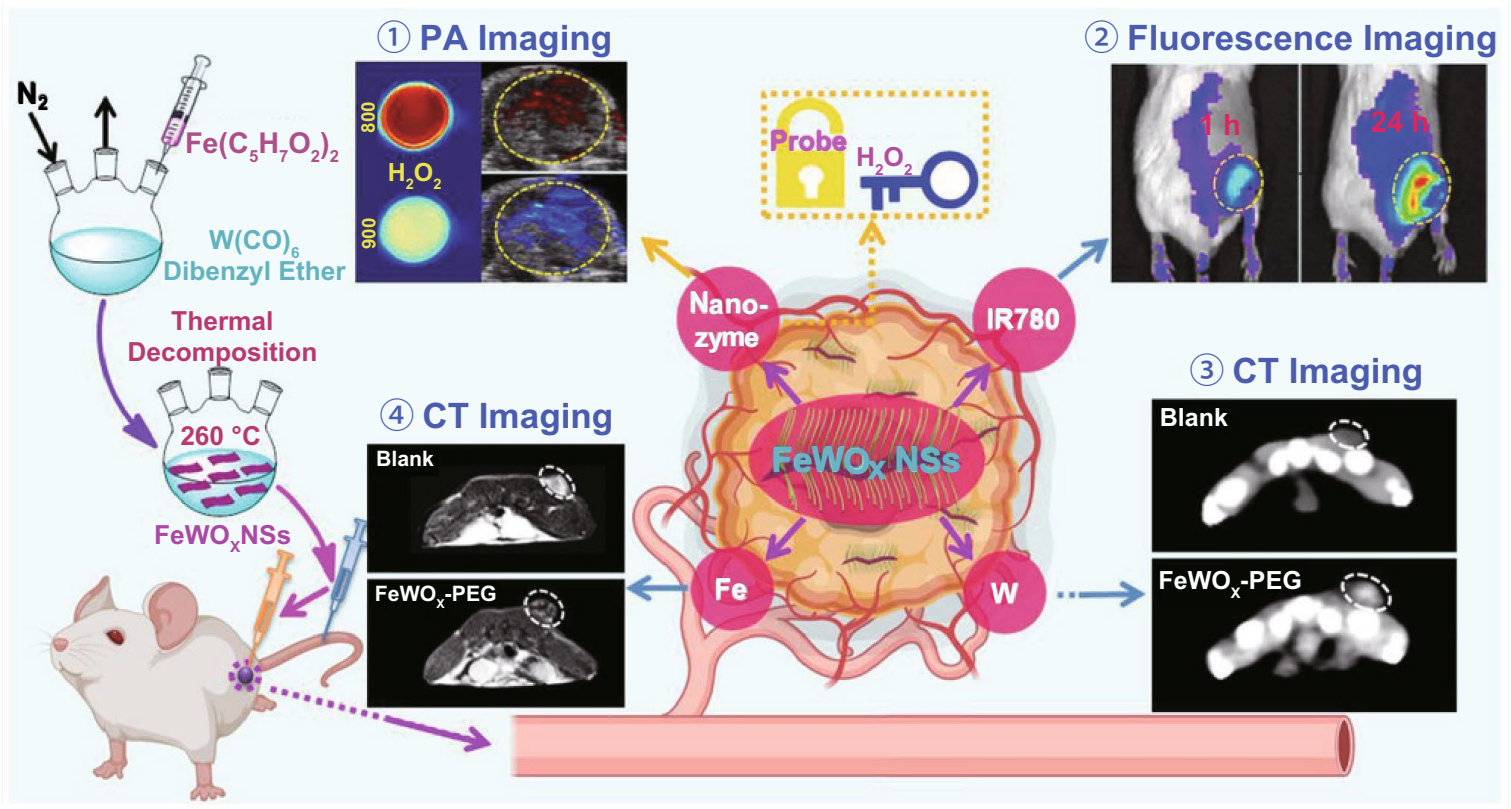
FeTIR nanoprobe with POD-like ability for multimodal tumor imaging. Reproduced with permission from Ref. [34]. Copyright WILEY–VCH GmbH 2020

As an example, Gong et al. synthesized a nanoprobe (FeTIR) in which TMB and a near-infrared dye (IR780) were coloaded on POD-like FeWO_X_ nanosheets for multimodal imaging [34]. Owing to the strong X-ray attenuation ability of the W element, the high MR contrast ability of the Fe element, and the superior fluorescence properties of IR780, the nanoprobe could be used for CT, MR, and fluorescence imaging of tumors (**Fig. ****9**). Owing to the high level of H_2_O_2_ in the tumor microenvironment, the loaded TMB in the FeTIR could be oxidized after reaching the tumor, leading to the remarkable increase in the PA signal at 900 nm (PA_900_). Combined with the loaded IR780 that had a PA signal at 800 nm (PA_800_) serving as the internal reference, the FeTIR nanoprobe could be used for ratio-metric PA imaging according to the ratio of PA_900_ to PA_800_.

## Nanozymes for Cancer Therapy

Nanozymes play versatile roles in cancer therapy. As nanomaterials, they can serve as drug carriers and passively target the tumor through the enhanced permeability and retention (EPR) effect. Similar to other nanomaterials, nanozymes can also be therapeutic agents such as photosensitizers, sonosensitizers, and radiosensitizers for diverse cancer treatments. On the other hand, with distinct enzyme-mimicking ability, nanozymes can be used for catalytic therapy and starvation therapy to directly kill tumor cells or inhibit tumor growth. Moreover, nanozymes can improve the anticancer performance by O_2_ supply and GSH depletion. Additionally, in situ activation of therapeutic agents can be realized under the catalysis of nanozymes. In this section, we emphasize the enzyme-mimicking property of nanozymes in cancer therapy (Table 6).

**Table 6:** Nanozymes for cancer therapy

Nanozyme	Activity	Substrate	Application	References
MoO_3−x_ NUs	CAT, OXD	H_2_O_2_, O_2_	CAT-OXD cascade catalytic therapy	[35]
P@Pt@P-Au-FA	CAT, GOx	H_2_O_2_, glucose, O_2_	O_2_ supply for enhanced starvation therapy and PDT	[36]
PCF-a	POD, GPx	H_2_O_2_, GSH	GSH depletion for enhanced ultrasound /NIR-promoted CDT	[37]
OxgeMCC-r SAE	CAT	H_2_O_2_	O_2_ supply for enhanced PDT	[38]
Cu-HCF	GSHOx, POD	GSH, H_2_O_2_	GSH depletion for enhanced cascade enzymatic therapy	[76]
Pt-decorated Zr-MOFs	CAT	H_2_O_2_	O_2_ supply for enhanced PDT	[83]
Ferrihydrite	CAT	H_2_O_2_	O_2_ supply for enhanced RT	[88]
SFO	CAT, GPx	H_2_O_2_, GSH	O_2_ supply and GSH depletion for enhanced CDT and PDT	[115]
Pt-CuS	CAT	H_2_O_2_	O_2_ supply for enhanced SDT	[117]
PtCu_3_ nanocages	POD, GPx	H_2_O_2_, GSH	GSH depletion for enhanced CDT and SDT	[118]
TMPAs	POD	TMB	Nanozyme-amplified NIR-II PTT	[120]
MnPcNPs	POD	H_2_O_2_	Catalytic therapy	[121]
Pd–C SAzymes	POD	H_2_O_2_	H_2_O_2_-supply catalytic therapy	[122]
DMSN-Au-Fe_3_O_4_	GOx, POD	Glucose, H_2_O_2_	OXD-POD cascade catalytic therapy	[125]
pyrite nanozyme	GSHOx, POD	GSH, H_2_O_2_	OXD-POD cascade catalytic therapy	[126]
mGPB	SOD, CAT, OXD, POD	O_2_^·−^, H_2_O_2_, glucose	SOD/CAT-OXD-POD cascade catalytic therapy	[127]
MnO_2_@PtCo	CAT, OXD	H_2_O_2_, O_2_	CAT-OXD cascade catalytic therapy	[129]
Nitrogen-doped porous carbon nanospheres	OXD, POD, SOD, CAT	O_2_, H_2_O_2_, O_2_^·−^	Combined catalytic therapy with reduced toxicity to normal tissues	[130]
MSNR@MnO_2_–Au	CAT, GOx	H_2_O_2_, glucose, O_2_	O_2_ supply for starvation therapy, RT, PTT	[132]
rMGB	CAT	H_2_O_2_	H^+^/O_2_ supply for enhanced PDT	[133]
Pt–TiO_2_	CAT	H_2_O_2_	O_2_ supply for enhanced SDT	[134]
Mn^2+^-doped Ag_2_Se quantum dots	CAT	H_2_O_2_	O_2_ supply for enhanced RT	[136]
Ir@liposome	CAT	H_2_O_2_	Controllable O_2_ supply for enhanced RT	[137]
IMSNs	CAT	H_2_O_2_	O_2_ supply for enhanced immunotherapy	[138]
IAA-loaded PNCNzyme	POD	IAA	Nanozyme-activated chemotherapy	[146]

### Catalytic Therapy and Cascade Catalytic Therapy

In catalytic therapy, cancer cells or tumors are inhibited by toxic reactive oxygen species (ROS) that are generated from the catalytic reactions mediated by enzymes. Inheriting the merits of both natural enzymes and nanomaterials, nanozymes have been promising systems for cancer catalytic therapy.

POD-mimicking nanozymes can promote the degradation of H_2_O_2_ to produce highly toxic hydroxyl radicals (·OH) for cell killing, As an example, Wang et al. synthesized MnPc nanoparticles (MnPcNPs) by the supramolecular assembling of manganese phthalocyanine (MnPc) [121]. The MnPcNPs with metal–N–C active centers had size-dependent POD-like activity. Their suitable size endowed the nanozymes with favorable tumor accumulation capability through the EPR effect. Moreover, MnPcNPs displayed higher catalytic activity in the acidic tumor microenvironment than in normal tissues and thus effectively killed cancer cells with the low side effect.

**Fig. 10:**
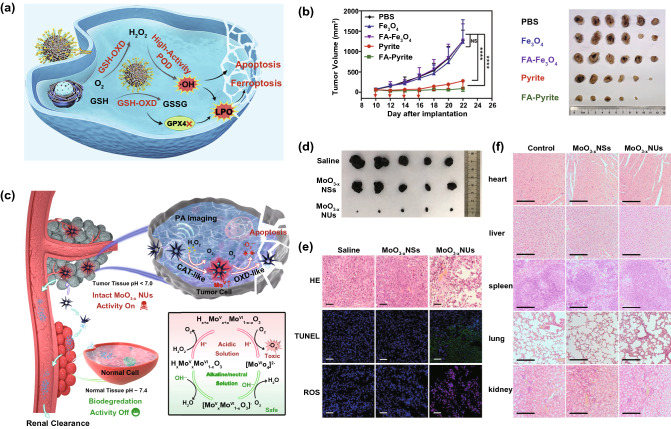
**a** Schematic illustration of the self-cascade pyrite nanozymes with both POD- and GSHOx-like abilities for cancer therapy. **b** In vivo anticancer performance of the pyrite nanozymes. Reproduced with permission from Ref.[126]. Copyright 2021, American Chemical Society. **c** Schematic illustration of the biodegradable MoO_3−x_ NUs with both CAT- and OXD-mimicking ability for cancer therapy. **d**–**e** In vivo anticancer performance of the pyrite nanozymes of MoO_3−x_ NUs. **f** Evaluation on the biosafety of MoO_3−x_ NUs. Reproduced with permission from Ref. [35]. Copyright 2019, American Chemical Society

To improve the therapeutic efficiency of POD-like nanozymes, increasing the H_2_O_2_ level in the tumor is a feasible way. For instance, both POD-like Pd–C single-atom nanozymes (SAzymes) and camptothecin (CPT) were encapsulated into agarose hydrogels for H_2_O_2_ self-supply based catalytic therapy [122]. The generated heat by Pd–C SAzymes upon NIR light irradiation could accelerate CPT release by promoting hydrogel degradation. Moreover, the released CPT could activate nicotinamide adenine dinucleotide phosphate oxidase, thus producing H_2_O_2_ for enhanced catalytic therapy. As OXDs and OXD-like nanozymes can catalyze the generation of H_2_O_2_, the combination of OXD- and POD-like nanozymes can lead to a cascade system for enhanced therapy [123, 124]. For this purpose, ultrasmall Au and Fe_3_O_4_ nanoparticles were packed into dendritic mesoporous silica (DMSN) to act as GOx- and POD-like nanozymes, respectively [125]. The loaded Au nanoparticles could promote the oxidation of glucose into H_2_O_2_ in the presence of dissolved oxygen, and the generated H_2_O_2_ was subsequently decomposed into ·OH with the help of the Fe_3_O_4_ nanoparticles. Compared to the single nanozyme-loaded DMSN, the Au and Fe_2_O_3_-coloaded DMSN showed significantly enhanced anticancer performance. Recently, Meng and coworkers prepared a self-cascade pyrite nanozyme that integrated both POD- and OXD-mimicking abilities into one nanoparticle [126]. As a POD mimic, the nanozyme with high H_2_O_2_ affinity possessed a three-order of magnitude higher catalytic activity than the classical Fe_3_O_4_ nanozyme and HRP, which could be beneficial for ·OH generation (Fig. 10a). Acting as GSHOx, the nanozyme could catalyze the oxidation of GSH accompanied by H_2_O_2_ formation, strengthening their anticancer ability (Fig. 10b). To further increase the efficiency of the OXD-POD cascade system, Chen et al. designed a cancer cell membrane-coated and GOx-carrying hollow mesoporous Prussian blue (mGPB) with multi-enzyme activities [127]. When reaching the tumor region through the homologous targeting ability of the cancer cell membrane, the mGPB with SOD/CAT-like catalytic activity could first use the intracellular O_2_^·−^ and H_2_O_2_ to generate O_2_. Then, the mGPB would further exhaust the produced O_2_ and the intratumoral glucose to generate plenty of H_2_O_2_ due to the presence of GOx in the system. Finally, the high level of H_2_O_2_ was transformed into massive ·OH under the catalysis of the POD-like mGPB.

Besides POD mimics, OXD-mimicking nanozymes could consume O_2_ to generate toxic O_2_^·−^, which is theoretically a feasible method for cancer treatment. However, due to the intrinsic hypoxic environment in the tumor, OXD-mimicking nanozymes are usually in combination with CAT or CAT mimics that can provide sufficient O_2_ for cascade catalytic therapy [128]. For example, Wang et al. prepared highly ordered MnO_2_@PtCo nanoflowers by the direct growth of MnO_2_ on PtCo nanoparticles [129]. Taking advantage of the CAT-like MnO_2_ and OXD-mimicking PtCo, the MnO_2_@PtCo nanoflowers could supply O_2_ to relieve the hypoxic conditions and boost ROS generation in the tumors, therefore exhibiting good therapeutic outcomes against cancer. Similarly, Hu et al. designed biodegradable molybdenum oxide nanourchins (MoO_3−x_ NUs) with both CAT- and OXD-mimicking abilities (Fig. 10c) [35]. In the tumor microenvironment, the MoO_3−x_ NUs behaved like CAT to catalyze the decomposition of H_2_O_2_, supplying abundant O_2_ for the following reaction. Substantial cytotoxic·O_2_^·−^ was produced to induce cancer cell apoptosis, thanks to the high OXD-like enzymatic activity of the nanozymes (Fig. 10d, e). In sharp contrast, MoO_3−x_ nanosheets (NSs) with low OXD-like activity had little influence on tumor growth. Interestingly, when placed in blood or normal tissues that have a neutral pH, MoO_3−x_ NUs would quickly be degraded with the loss of catalytic activity, endowing the nanozymes with suitable safety (Fig. 10f).

Additionally, OXD- and POD-mimicking nanozymes can be integrated into a system for combined catalytic therapy. Fan and coworkers reported the synthesis of nitrogen-doped porous carbon nanospheres with multiple enzyme-like activities [130]. Once localized in an acidic environment such as lysosomes, the nanozymes could behave like OXD and POD, transferring O_2_ and H_2_O_2_ into toxic O_2_^·−^ and ·OH, respectively. Interestingly, the nanozymes also performed CAT- and SOD-like activities under a neutral pH environment, which might reduce their cytotoxicity to normal tissues.

### Starvation Therapy

Starvation therapy which cuts off energy supply for inhibiting tumor growth has become an alternative cancer treatment. As a consequence of their rapid metabolism and proliferation, cancer cells usually need more energy than normal ones, making them more sensitive to energy. Hence, the depletion of energy can be used to inhibit tumor growth. As the most important source of energy in a living system, glucose can be oxidized into gluconic acid and H_2_O_2_ by GOx or GOx-like nanozymes with the help of O_2_, which can be used for starvation therapy [131]. However, the tumor has a hypoxic microenvironment, which weakens the activity of O_2_-dependent GOx and its mimics. To overcome the limitation, O_2_-supply enzymes or nanozymes are often used in starvation therapy.

**Fig. 11:**
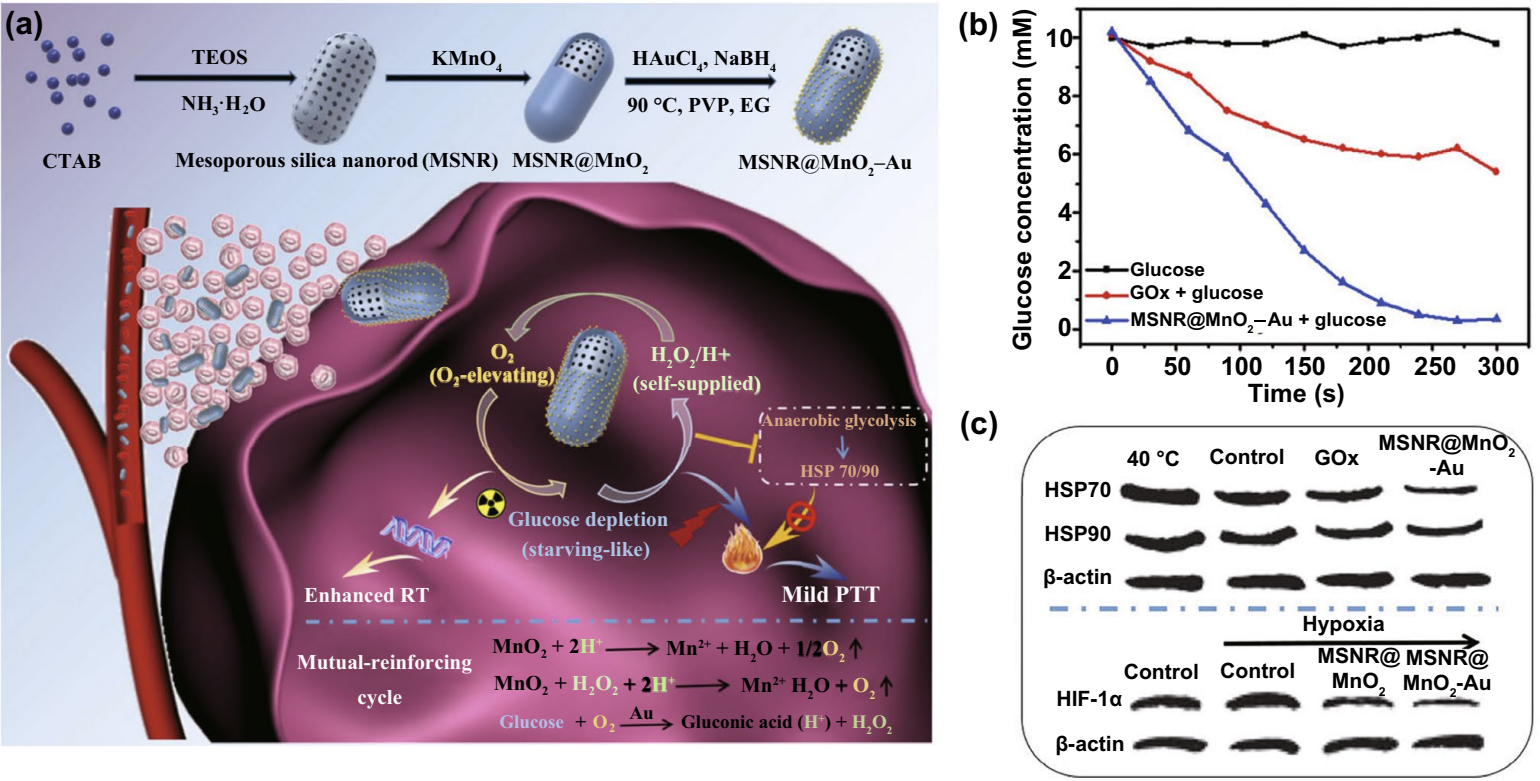
**a** Schematic illustration for the preparation of MSNR@MnO_2_–Au for RT and starvation-promoted mild PTT. **b** Glucose consumption performance of MSNR@MnO_2_–Au. **c** Western blot images of HSP70, HSP90, and hypoxia-inducible factors-1α (HIF-1α) expression in MCF-7 cells treated with different formulations. Reproduced with permission from Ref. [132]. Copyright 2020, Tsinghua University Press and Springer-Verlag GmbH Germany, part of Springer Nature

Liu et al. designed dual-nanozyme-based nanosystems (termed P@Pt@P-Au-FA) composed of platinum nanoparticles, AuNPs, and porphyrin metal–organic frameworks (PCN) [36]. The CAT-like Pt nanoparticles could catalyze the generation of O_2_ for promoting the oxidation of glucose by GOx-like Au nanoparticles for starvation therapy. Yang et al. designed biomimetic mesoporous silica nanorod (MSNR)@MnO_2_–Au nanozymes, in which MnO_2_ and Au nanoparticles had CAT- and GOx-like activity (Fig. 11a) [132]. Under the catalysis of MnO_2_, the H_2_O_2_ in the tumor was degraded into O_2_, which could enhance the Au-catalyzed glucose oxidation (Fig. 11b) and the radiotherapeutic effect. Moreover, acute glucose consumption could not only be used for starvation therapy, but also downregulate the expression of heat shock proteins including HSP70 and HSP90 (Fig. 11c), realizing starvation-promoted mild photothermal therapy (PTT). As a result, the nanozymes exhibited a significant tumor inhibition effect combined with PTT and RT.

### Oxygen Supply

Oxygen plays an important role in different cancer treatments such as photodynamic therapy (PDT), SDT, RT, and even immunotherapy. Nevertheless, the intrinsic hypoxia in the tumor hinders the efficacy of these treatments. Nanozymes with CAT- or SOD-like activity can catalyze the generation of O_2_ and alleviate the hypoxia tumor microenvironment, and thus have been broadly used.

**Fig. 12:**
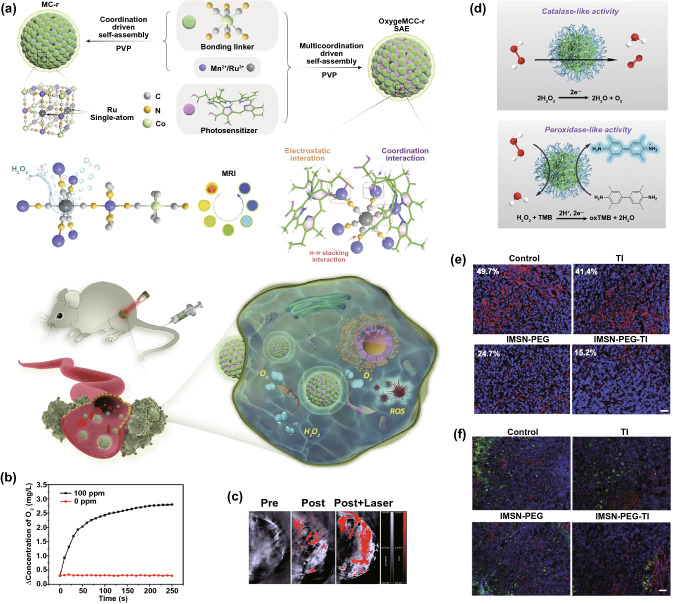
**a** Schematic illustration of the OxgeMCC-r nanozyme with CAT-activity to oxygen self-supply PDT. Reproduced with permission from [38]. Copyright Nature Publishing Group 2020. **b** O_2_ production ability of Pt-CuS in the presence of H_2_O_2_. **c** Blood oxygen saturation of tumors after different treatments. Reproduced with permission from Ref. [117]. Copyright 2019, American Chemical Society. **d** Schematic presentation for the CAT- and POD-like activities of IMSNs. **e** Immunofluorescence images of HIF-1α in the tumor slides. HIF-1α and cell nuclei were stained by red and blue fluorescence, respectively. **f** Immunofluorescence images of macrophages in tumor tissues. M1 and M2 macrophages were stained by red and green fluorescence, respectively. Reproduced with permission from Ref. [138]. Copyright 2020, WILEY–VCH GmbH

For example, in type II PDT, O_2_ is converted into highly toxic singlet oxygen (^1^O_2_) with the help of photosensitizers under light/laser irradiation, which can be used for tumor therapy. For improving the efficiency of PDT, Pt nanozymes were decorated on porphyrinic Zr-based metal–organic frameworks (Zr-MOFs) via in situ reduction [83]. The loaded Pt nanozymes with high CAT-like activity could induce the degradation of H_2_O_2_ into O_2_ in the tumor, accelerating the generation of ^1^O_2_ to kill cancer cells after laser irradiation. Similarly, Wang and coworkers integrated single-atom ruthenium and photosensitizer chlorin e6 (Ce6) into Mn_3_[Co(CN)_6_]_2_ metal–organic frameworks to form OxgeMCC-r single-atom enzyme (SAE), in which single-atom ruthenium served as the active catalytic site (Fig. 12a) [38]. Under the catalysis of the CAT-like nanozymes, the endogenous H_2_O_2_ in the tumor could be converted into O_2_, leading to the increase in ROS generation and cancer cell death after PDT. To further accelerate the O_2_ generation, Yang et al. reported a biomimetic hybrid nanozyme (named rMGB) containing natural GOx and MnO_2_ nanozymes [133]. The encapsulated GOx could catalyze the oxidation of glucose into gluconic acid, depleting energy for starvation therapy and generating H^+^ to improve the catalytic efficiency of CAT-like MnO_2_. Thus, the H^+^/O_2_ self-supply rMGB realized enhanced starvation therapy and PDT against hypoxic tumors.

Similar to PDT, SDT, in which ultrasound is used to activate sonosensitizers to generate toxic ROS for cancer treatment, is also O_2_ concentration-dependent. To mitigate the tumor hypoxia for improved SDT, Liang and coworkers designed a Pt-CuS Janus system composed of hollow CuS and Pt nanozymes [117]. The hollow CuS showed a large inner cavity for loading sonosensitizers. The deposition of Pt not only enhanced photothermal performance, but also provided CAT-like ability for catalyzing the production of O_2_ that can overcome tumor hypoxia for enhanced cancer therapy (Fig. 12b). More interestingly, the heat generated by laser irradiation of Pt-CuS could improve the enzymatic activity of Pt and accelerate the generation of O_2_, which further improved the anticancer efficiency of SDT (Fig. 12c). In recent work, they developed a Pt–TiO_2_ heterostructure with an oxygen-deficient layer as a bilaterally enhanced sonosensitizer [134]. The hollow cavity of TiO_2_ could serve as a reservoir to carry doxorubicin, a chemotherapeutic agent and sonosensitizer. The Pt nanoparticles behaved like CAT to promote the decomposition of H_2_O_2_, providing sufficient O_2_ for subsequent SDT.

In RT, external beam radiation or internal radiation is locally administrated at the tumor site to destroy cancer cells by generating ROS. Thus, the radiotherapeutic efficacy is affected by the level of available O_2_ within the tumor [135]. However, the low O_2_ level in the tumor microenvironment usually leads to cancer radio-resistance or failure of RT. To overcome this dilemma, Wang et al. doped Mn^2+^ into second near-infrared (NIR-II)-emitting Ag_2_Se quantum dots to endow the system with CAT-like activity [136]. By the catalysis of Mn^2+^ in the quantum dots, H_2_O_2_ was decomposed into O_2_. Owing to the surface modification of the tumor-targeting arginine-glycine-aspartate unit, the nanozymes could be accumulated in the tumor region. Combined with the radiosensitive activity of the element Ag, the nanozyme realized enhanced imaging-guided RT of tumors. In recent work, Zhang et al. found that ferrihydrite, especially 2-line ferrihydrite, possessed the strongest CAT-like activity among the main forms of iron oxide nanomaterials, without POD- or SOD-like activities [88]. Combined with its excellent biosafety and steady activity, the ferrihydrite showed a great potential for enhanced cancer RT by producing O_2_ and alleviating hypoxia. To control the O_2_ generation in the tumor, ultrasmall iridium nanocrystals with CAT-like activity were encapsulated into liposomes, endowing the system with near-infrared light responsivity [137]. Under NIR light irradiation, the obtained Ir@liposome would show increased catalytic activity to trigger O_2_ generation, which together with the intrinsic radio-sensitizing ability of Ir would enable remarkably enhanced cancer RT.

In addition to participating in ROS generation during PDT, SDT, and RT, O_2_ can also mediate the immune system. The tumor hypoxia impairs anticancer immunity by altering the functions of immune cells or by increasing cancer resistance to immune effectors [28]. To reverse the tumor immunosuppressive environment, iron manganese silicate nanozymes (IMSNs) were fabricated. Loading with TGF-β inhibitor (TI) after polyethylene glycol (PEG) modification formed IMSN-PEG-TI (Fig. 12d) [138]. The nanozymes exhibited CAT-like activity to catalyze O_2_ generation to overcome tumor hypoxia and regulate the immune microenvironment (including increasing the level of M1 macrophages) (Fig. 12e, f). In addition, the nanozymes also had POD-like activity to decompose both the endogenous H_2_O_2_ and the H_2_O_2_ generated by M1 macrophages into ·OH for catalytic therapy.

### GSH Depletion

As we discussed above, GSH, as the detoxification and antioxidant agent by deactivating radicals and reactive oxidants, is responsible for cancer drug resistance and severely affects the cancer treatment outcome. To overcome the limitation, a large number of nanomaterials with GSH-depleting ability have been developed to enhance the anticancer efficiency of different ROS-generated therapeutics such as chemotherapy [139], RT [140], PDT [141], chemodynamic therapy (CDT) [142], and SDT [143]. For nanozymes with GPx- or GSHOx-mimicking ability, they can catalyze the oxidation of GSH, thus consuming the intracellular GSH for enhanced cancer therapy.

**Fig. 13:**
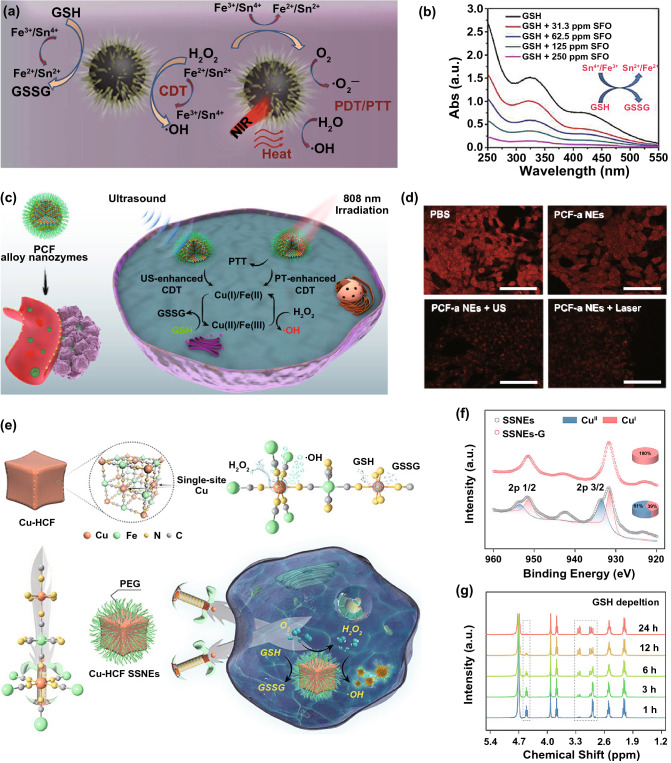
**a** Schematic illustration of the synergistic therapy mechanism for SFO. **b** Concentration-dependent GSH depletion ability of SFO. Reproduced with permission from Ref. [115]. Copyright 2020, WILEY–VCH GmbH. **c** Schematic illustration of PCF-a nanozyme with POD- and GPx-like activities for dual-modal cancer therapy. **d** Intracellular GSH levels in cancer cells with different treatments. Reproduced with permission from Ref. [37]. Copyright 2021, American Chemical Society. **e** Schematic illustration of Cu-HCF single-site nanozymes (SSNEs) for tumor-specific cascade enzymatic therapy. **f** Cu 2p X-ray photoelectron spectroscopy results of SSNEs and SSNEs-G. “SSNEs-G” represents GSH-pretreated SSNEs. **g** ^1^H nuclear magnetic resonance (NMR) spectra of GSH at different reaction times in the presence of SSNEs. Reproduced with permission from Ref. [76]. Copyright 2020, WILEY–VCH GmbH

For example, Zhong et al. reported the synthesis of multifunctional PtCu_3_ nanocages that could simultaneously act as sonosensitizers as well as POD- and GPx-like nanozymes [118]. As sonosensitizers and POD-like nanozymes, PtCu_3_ nanocages could generate massive ROS to kill cancer cells, especially when exposed to ultrasound. Moreover, the intratumoral GSH could be depleted by the GPx-like PtCu_3_ nanocages, thus weakening the GSH-mediated ROS-scavenging capacity of cancer cells. In another work, Feng et al. introduced the SnFe_2_O_4_ (SFO) nanozymes with both CAT- and GPx-like activities for synergistic therapy (Fig. 13a) [115]. Like CAT, the nanozymes could trigger the degradation of H_2_O_2_ into O_2_ to meliorate the tumor hypoxia, which is beneficial for PDT. In addition, SFO nanozymes could also generate ·OH, not only for CDT but also for consuming the GSH to relieve the antioxidant capability of the tumors (Fig. 13b). Jana et al. reported the preparation of ultrasmall trimetallic (Pd, Cu, and Fe) alloy nanozymes (PCF-a) with dynamic active-site synergism [37]. The presence of Cu and Fe active sites endowed the nanozymes with POD-mimicking activity to produce ·OH radicals at neutral pH, and the nanozymes showed enhanced ROS generation when exposed to NIR and ultrasound (Fig. 13c). Furthermore, PCF-a exhibited the GPx-like property to exhaust GSH (Fig. 13d), protecting the generated ROS from being consumed by the GSH of cancer cells. Recently, Wang et al. synthesized copper hexacyanoferrate (Cu-HCF) nanozyme with active single-site copper for cascade enzymatic therapy (Fig. 13e) [76]. Their GSHOx-like activity enabled the Cu-HCF single-site nanozymes to deplete intracellular GSH and convert single-site Cu^II^ species into Cu^I^ (Fig. 13f, g). The generated Cu^I^ could trigger the subsequent decomposition of H_2_O_2_ into ·OH. Thus, the Cu-HCF with both GSHOx- and POD-like activities could accumulate massive ROS in the tumor for efficient cancer therapy.

### Activation of Therapeutics

Conventional therapeutic agents exhibit cell-killing ability in all parts of the body where they accumulate, causing undesirable side effects. The employment of activable therapeutics is an effective method to overcome the problem. Owing to their enzymatic property, nanozymes can catalyze the activation of the therapeutic agents for cancer therapy [144].

HRP, as a kind of POD, can catalyze the oxidation of indole-3-acetic acid (IAA) and its derivatives into cytotoxic species, which can inhibit cancer cell growth effectively [145]. Inspired by this property, a phosphorous- and nitrogen-codoped porous hollow carbon sphere nanozymes (PNCNzyme) with POD-mimicking activity in acidic conditions was developed, and IAA was loaded via π–π stacking interactions [146]. Furthermore, folate was introduced on the surface of the nanozyme to enhance its tumor-targeting ability and promote its endocytosis by folate receptor-overexpressed cancer cells. When reaching the lysosomes with an acidic environment, the loaded IAA was activated by the PNCNzyme to generate sufficient free radical intermediates, causing the apoptosis of cancer cells.

**Fig. 14:**
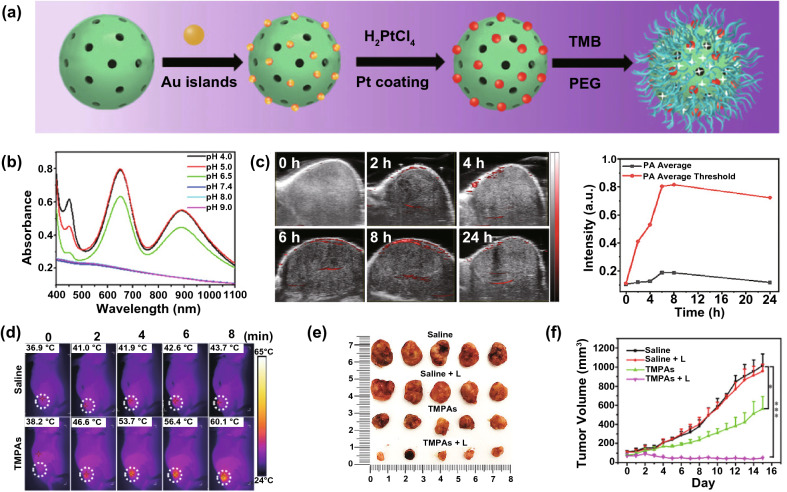
**a** Schematic illustration showing the synthetic routine of TMPAs. **b** UV–Vis–NIR absorption spectra of TMPAs at different pH values. **c** In vivo PA images of tumor-bearing mice after intravenous injection of TMPAs, and the corresponding time-dependent intensity of PA signals. **d** IR thermal images of tumor-bearing mice injected with saline or TMPAs after laser irradiation. **e**, **f** In vivo antitumor performance of different treatments. Reproduced with permission from Ref. [120]. Copyright 2021, WILEY–VCH GmbH

Recently, Ma and coworkers rationally designed a nanoreactor (TMB/MOF/PtAu-PEG, TMPAs) in which TMB and PtAu nanozymes were encapsulated into a metal–organic framework decorated with PEG (Fig. 14a) [120]. The nanoreactor stayed inactivated during blood circulation and healthy tissues. When arriving at the acidic tumor microenvironment, the loaded PtAu nanozymes could act as PODs and promote the production of ROS in the presence of the endogenous H_2_O_2_ for catalytic therapy. Moreover, the nanoreactor could catalyze the oxidation of TMB with no NIR absorption into cationic TMB having broadband NIR absorption (Fig. 14b), which was beneficial for tumor-specific PA imaging (Fig. 14c) and PTT (Fig. 14d–f).

## Conclusions and Perspectives

To date, a large number of nanozymes have been developed to mimic natural enzymes such as POD, OXD, CAT, and SOD. The activities of nanozymes can be modulated by altering their physicochemical properties such as composition, size, morphology, crystal face, surface modification, valence, and active site [147]. By integrating the properties of natural enzymes and nanomaterials, nanozymes own the advantage of superior catalytic activity, low production cost, high stability, good controllability, and multifunctionality, making them suitable for cancer diagnoses including cancer-related gene/molecule/cell detection and tumor imaging. Moreover, nanozymes can serve as drug-free formulations to directly kill cancer cells, remodel the tumor environment for enhanced cancer therapy, and/or catalyze the in situ formation of therapeutic agents. Although significant progress has been made in recent years, there is still room for advancement in the research of nanozymes in cancer theranostics. Here, we put forward several critical issues and research directions that can be studied in the future to increase the possibility of clinical translation of nanozymes.

First of all, besides mimicking the activity of oxidoreductases, nanozymes can act as hydrolases [148–151]. For instance, Guan et al. prepared the ceria/polyoxometalate nanohybrid that not only behaved like protease to degrade amyloid-β peptides but also acted as SOD to reduce ROS [148]. Chen and coworkers constructed a DNase-mimetic artificial enzyme (DMAE) composed of AuNPs with multiple cerium (IV) complexes on the surface of Fe_3_O_4_/SiO_2_ core/shell particles [149]. Both genomic DNA and extracellular DNA could be effectively cleaved by DMAE. The hydrolase-like nanozymes can be employed to activate probes or prodrugs for nanozyme-triggered cancer theranostics. Moreover, since some natural hydrolases such as *L*-asparaginase (AspNase) show intrinsic anticancer ability [152], it is envisioned to develop AspNase-like nanozymes as chemotherapeutics for directly inhibiting tumors. Second, natural enzymes are classified into seven types based on their activities including oxidoreductases, transferases, hydrolases, lyases, isomerases, ligases, and translocases. More efforts should be devoted to developing novel nanozymes with diverse enzymatic activities and employing them for cancer diagnosis and therapy. Third, most nanozymes have multiple enzyme-mimicking capabilities, which may create a competitive relationship. For example, as both CAT and POD can catalyze the decomposition of H_2_O_2_, the nanozymes with both CAT- and POD-like abilities may have reduced enzymatic activities as compared to those with a single enzyme-mimicking ability. To solve the problem, it is expected to find general principles to guide the design of nanozymes and develop more nanozymes with excellent single enzyme-mimicking ability. Fourth, nanozymes that mimic multiple enzymes can realize cascade reactions for cancer diagnosis or cascade therapy. It is still of great challenge to rationally design the nanozymes with excellent multi-enzyme-like activities. Fifth, to further improve the stability, biocompatibility, selectivity, and/or subcellular/tumor-targeting ability of nanozymes, surface modification is commonly used [153]. However, it may hinder the interaction between the surface of nanozymes and substrates, leading to unsatisfying catalytic performance. Thus, the surface modification methods should be optimized and it is needed to develop a strategy that can minimize the influence of surface modification on the activity of nanozymes. Sixth, only a few recent studies utilize nanozymes to “turn on” the therapeutic agents. More nanozyme-based activable systems should be developed. Seventh, for efficient catalytic reactions with high selectivity and effective cancer treatment with negligible side effects, the activity of nanozymes should be controllable. Up to now, a number of nanozymes have been designed, which are sensitive to different stimuli such as pH, light, thermal, and ultrasound. More nanozyme systems with high controllability are highly desirable. Eighth, owing to the presence of tumor heterogeneity, it is still challenging to design a nanozyme-based system for the accurate diagnosis and treatment of different types of tumors. Ninth, drug resistance is one of the main reasons for the failure of cancer chemotherapy. Although specific nanozymes have been demonstrated to kill chemotherapeutics-resistant cancer cells [154], it remains unknown whether the nanozyme-based treatment leads to the drug resistance. Tenth, some nanozymes are highly stable and hard to be excreted from the body, while others are easy to be metabolized and also prone to inactivate in the living system. As a result, the design of nanozymes for in vivo applications should balance stability and biosafety. Finally but not least, the toxicology and biosafety data of nanozymes are still lacking. Before future clinical uses, the nanozymes should meet the demands of biosafety with high enzymatic efficiency. It is worthwhile to evaluate the long-term biocompatibility and catalytic efficiency of nanozymes. Thus, this review article is expected to stimulate the further development of the nanozyme field.
